# Effectiveness of sexual health counseling based on mindfulness approach on sexual satisfaction in women suffering from infertility: An RCT

**DOI:** 10.18502/ijrm.v21i2.12805

**Published:** 2023-03-08

**Authors:** Sara Hosseini Nejad, Mahshid Bokaie, Seyed Mojtaba Yassini Ardekani

**Affiliations:** ^1^College of Nursing and Midwifery, Shahid Sadoughi University of Medical Sciences, Yazd, Iran.; ^2^Research Center for Nursing and Midwifery Care, Shahid Sadoughi University of Medical Sciences, Yazd, Iran.; ^3^Department of Psychiatry, Research Center of Addiction and Behavioral Sciences, Shahid Sadoughi University of Medical Sciences, Yazd, Iran.

**Keywords:** Infertility, Women, Sexual satisfaction, Mindfulness, Psychology.

## Abstract

**Background:**

The infertility phenomenon affects physiological and psychosocial aspects. Sexual counseling can improve sexual and marital satisfaction.

**Objective:**

This study aimed to determine the effectiveness of sexual health counseling based on the mindfulness approach on sexual satisfaction of women suffering from infertility.

**Materials and Methods:**

This randomized clinical trial study was conducted on 44 women suffering from infertility referred to the Yazd Reproductive Science Institute, Yazd, Iran. Women were randomly divided into 2 groups. The intervention group (n = 22) received 8 sessions of sexual counseling, while control group (n = 22) received routine care. The data collection tool was the sexual satisfaction questionnaire.

**Results:**

The mean age of women was 30.05 ± 4.9 yr, infertility duration was 6.83 ± 4.27 yr, and the mean duration of marriage was 8.3 ± 4.01 yr. The mean score of sexual satisfaction of women suffering from infertility in the intervention group was 62.9 ± 7.32, 71.6 ± 5.95, and 70.9 ± 6.26, before the intervention (baseline), after intervention (8 ${}^{\text{th}}$ wk), and follow-up (12 ${}^{\text{th}}$ wk) respectively. The mean score of sexual satisfaction of women suffering from infertility in the control group was 63.3 ± 6.82, 64.2 ± 7.93, and 62.25 ± 7.99, at baseline, 8 ${}^{\text{th}}$ wk later, and after follow-up (12 ${}^{\text{th}}$ wk), respectively. Sexual satisfaction scores increased before and one month after the intervention in the counseling group, and this difference was statistically significant (p < 0.001).

**Conclusion:**

Mindfulness-based sexual health counseling programs may improve sexual satisfaction in women suffering from infertility in the intervention group.

## 1. Introduction

Despite the variations in attitudes toward sexuality in recent centuries, the importance of fertility is still maintained in the human mind, with one being a factor in strengthening marital life (1). The occurrence of infertility for females has increased due to several reasons such as late marriage and late childbirth (2). In contrast to fertility, there is another important aspect called infertility, defined as the inability to have a child after 1 yr of continuous sexual activity without using contraceptive methods (3). Epidemiological studies show that infertility affects about 10-15% of couples in the United States and 20% of the Western population (4, 5). In Iran, this amount reaches to more than 3 million infertile couples (6).

Infertility is a multifactorial problem, and many couples do not have enough knowledge and skills to manage this problem properly. In recent years, considerable attention has been paid to the role of psychological aspects of infertility, and medical knowledge, suggests the link between infertility and psychological factor (7). The correlation analysis discovered significant positive correlations between sexual dissatisfaction and infertility-related and sexual concerns in couples (8).

In Iranian infertile couples, the most common psychological and emotional problems are dissatisfaction, frustration, anxiety, and fear (9). Stress, depression, low self-esteem, marital dissatisfaction, sexual dissatisfaction, impaired marital quality, decreased intimacy, fear of ending a marital relationship, helplessness, and clinical depression manifestations have been reported as psychological consequences of infertility (10, 11). Many studies have investigated the effects of infertility on sexual satisfaction and self-esteem (12), sexual function (13), and the social effects of infertility among couples (14). Sexual satisfaction is every person's judgment about the sexual behavior they enjoy, and it is largely affected by the consequences of infertility, such as having sex with failure to conceive. Decreased sexual satisfaction for any reason has many negative consequences. During infertility treatment, 50-60% of couples reported a marked decrease in sexual satisfaction (15). Sexual counseling can affect the quality of sexual relations, lead to increased satisfaction in sexual relations between couples, and may increase their enjoyment (16).

All processes play a role in the relationship between specific aspects of infertility-related sexual satisfaction. The authors' suggest psychosocial support for couples experiencing infertility (17).

Psychological treatments, along with infertility treatment programs, increase mental health, make infertile people more resistant to stress, increase the effectiveness of infertility treatments, and pursue infertile people for follow-up treatment. Mindfulness-based cognitive therapy is a recent development in cognitive therapy that is a short-term, structured intervention based on Kabat-Zinn's (1990) mind-based stress reduction model and incorporating cognitive therapy principles (15).

Due to the increasing prevalence of infertility among women, and its psychological impacts, such as decreased sexual satisfaction, the use of psychological treatment approaches is particularly important for sexual satisfaction. Regarding the limited counseling interventions in sexual health with a mindfulness approach in infertile women, this study aimed to determine the effect of mindfulness sexual health counseling on the sexual satisfaction of these women in Yazd.

## 2. Materials and Methods

### Study design and setting

This randomized clinical trial study with the control group (baseline, after the intervention, and follow-up) was done on women suffering from infertility referred to Yazd Reproductive Sciences Institute, Yazd, Iran from October 2019 to Febuary 2020.

The sample size was calculated to be 44 (22 in each group), considering the significance level of 5%, the power of 80%, and the type II error of 0.20. 


$$n=\frac{2\times S^{2}\times p\;\left(Z_{1-\frac{\alpha }{2}}+Z_{1-\beta }\right)}{\mu^{2}\times d}$$

In this formula, 1-α and 1-β are the reliability and test power levels, respectively, and were considered equal to 0.95 and 0.80. As a result, Z_ (1-α / 2) and Z_ (1-β) of the normal distribution table was 1.98 and 0.58, respectively.

From 152 eligible women, 44 candidate participated in this study. We used “random allocation” using 2 steps, first generating the random sequence by a computer program, then minimizing the effect of bias, the random allocation sequence remained concealed from midwives who enrolled patients in the study. Participants were randomly divided into 2 groups with computer-generated random numbers.

Each woman was assigned a number between 1-44, which was done by referring to the online randomization site. Currently, there are 3 random allocation models, and considering that this model is in the form of 2 groups, the first-generation randomization model was used, and each sample was placed in one group by the site. Due to duration of intervention (8 face-to-face sessions weekly), many women did not agree to participate in the study. Some people did not like to do mindfulness homework.

**Figure 1:**
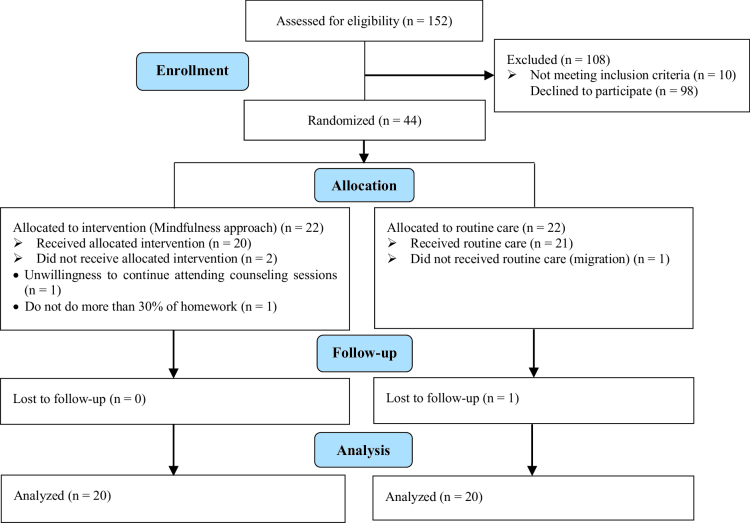
Consort flow diagram.

Overall, 44 participants were selected through convenience sampling with regard to inclusion and exclusion criteria. The control group was placed on the waiting list due to ethical considerations. Counseling sessions were arranged upon their request. Masking was not done because the participants were aware of the sexual counseling sessions. In addition to women, the spouse's consent was obtained due to ethical considerations in Iran. Because at first, the researchers said that the 2 groups have the same chance of being placed in the intervention and control groups, and most of them wanted to be in the intervention group, they were told that at the end of the intervention, mindfulness counseling would be done for them. Consort was shown in figure 1.

Inclusion criteria included: having been diagnosed with infertility for at least 1 yr; being the only wife of a man; being in their first marriage; living in Yazd, Iran; having reading and writing skills; being between the ages of 22 and 49; not attending tentative sessions or other psychological interventions concurrently during the study; and being willing to participate in the study.

Exclusion criteria included: history of mental illness according to the patient's self-reporting; taking psychiatric medications; having chronic diseases; addiction; and husband's diabetes.

All participants completed a sociodemographic (18) and sexual satisfaction (19) questionnaire. The questionnaire consisted of 25 questions, with 5-point scale answers and a Likert scale from 1-5. This questionnaire had acceptable validity in Iran. Cronbach's alpha for all items was above 0.70. Confirmatory factor analysis established the final factor construct of this questionnaire (20).

### Intervention

The sociodemographic and sexual satisfaction questionnaires were completed by all of the participants before the intervention (baseline), after the intervention (8 ${}^{\text{th}}$ wk), and during the 4 wk follow-up (12 ${}^{\text{th}}$ wk).

The first author held 8 (75-min) sexual satisfaction counseling program sessions with a mindfulness approach for the intervention group. She had a certificate of ability to perform mindfulness approach under the supervision of the 2 ${}^{\text{nd}}$ and 3 ${}^{\text{rd}}$ authors. This counseling session was conducted based on the content of previous related studies, and opinions of experts, including a Ph.D. in sexual and reproductive health and a Psychiatrist.

The content of the sessions is programed in table I. Weekly, 8 sessions (75 min) of mindfulness counseling were held for the intervention group (9), and routine care was performed for the control group. At the end of the study, 4 brief counseling sessions were held for the control group, due to ethical considerations. The questionnaire was filled by participants under M.Sc. student supervision. Counseling classes were held in a suitable room at the Institute of Reproductive Medicine, Yazd, Iran.

**Table 1:** The content of mindfulness-based sexual counseling sessions


**Session**	**Content of meetings**	**Homework**
**1**	Explain the research and the number of sessions Explain about relationship Distribution of questionnaires Doing the first mindfulness exercise (eating raisins with mindfulness) Ask some questions about sexual issues after infertility	Thinking about the sexual concepts mentioned in class Study of the booklet given by the researcher Do one of your daily activities, such as practicing raisin eating, and record it on your homework sheet
**2**	Talking about sexuality after an infertility diagnosis, and infertility challenges Focus on the body and discuss her experience and body image Discuss the difference between thoughts and feelings after infertility Breathing training with the presence of mind	Record pleasant events of thoughts and feelings Doing one of the daily activities with the presence of the mind, such as washing dishes Perform breathing with the presence of mind and focus on the body, and record in the homework
**3**	Thoughts and feelings during sex, fantasy Breathe with the presence of mind for 3 min Practice meditation in a sitting position Practice seeing and hearing Expressing the connection between unpleasant events after infertility	Focus on the body Practice 3 min of breathing space Make a list of unpleasant events, write down thoughts and feelings, and bodily sensations related to them and record it on the homework sheet
**4**	Talk about clients' sexual experiences, relationships, spirituality and intimacy Perform 3 min of breathing space 5 min of practice of seeing and hearing	Focus on the body and practice 3 min of breathing space, practice 5 min of seeing or hearing and recording on homework
**5**	Talk about clients' misconceptions about sex, childbearing Perform 3-min breathing space - coping	Performing sitting reflection and breathing space for 3 min, performing breathing space for 3 min - coping and recording in the homework sheet
**6**	Talk about clients' experiences for better sex Sitting reflection Awareness of breathing, body, sound, and then thoughts Practice breathing space for 3 min	3-min breathing space, 3-min breathing space - confrontation and recording in the homework sheet
**7**	Reflection sits along with awareness of breathing, body, sound, and then thoughts Practice observing the relationship between activity and mood Make a list of enjoyable activities, 3 min of breathing space	Walking with the presence of mind 3-min breathing space, 3-min breathing space - confrontation and recording in the homework sheet
**8**	Talk about clients' experiences Exercise body check Distribution of the questionnaire	Advise to continue home exercises

### Ethical considerations

This study was based on the Master's Degree in Midwifery Counseling with Code 6425 and it was approved by the Ethical Committee of Shahid Sadoughi University of Medical Sciences, Yazd, Iran (Code: IR.SSU.REC.1398.003) and the project registration at the clinical trial site. In this study, ethical issues such as informed consent, privacy, confidentiality and anonymity were considered. Written consent was obtained from all of them.

### Statistical analysis

After filling the questionnaires, the Shapiro-Wilk normality test was used to determine the quantitative normal distribution. An independent *t* test was used to examine quantitative variables such as age, number of pregnancies, duration of marriage and infertility duration, and Chi-square test and Fisher's exact test were used to compare qualitative data. To measure sexual satisfaction repeated measurement- ANOV and Bonferroni post hoc test were used.

Then, analysis was done using SPSS 17 software (Statistical Package for Social Sciences version 17.0, Chicago, Illinois, USA) and the significance level was considered < 0.05.

## 3. Results

This study aimed to determine the effectiveness of sexual health counseling based on the mindfulness approach to sexual satisfaction of women suffering from infertility. From 44 of them statistical analysis was done on 40 women.

The Shapiro-Wilk test showed that the data were normal. Results showed that the mean score of age in the intervention group was 30.65 ± 4.76 and in the control group was 29.45 ± 5.10 (p = 0.44). The duration of marriage in the intervention groups and the control group were 8.9 ± 4.74 and 7.7 ± 3.11 yr, respectively. The duration of infertility in the intervention group was 7.2 ± 5.26 yr in the control group, it was 6.47 ± 3.08 yr (p = 0.35). Mean score of the number of pregnancies in the intervention group was 1.8 ± 0.41 and in the control group was 1.85 ± 0.36 (p = 0.68). According to the independent *t* test, the results showed that the mean score of age, duration of infertility, the number of pregnancies, there are no statistical differences between the 2 groups. The majority of women in the intervention and the control group were housewives (p = 0.48). The Chi-square test, and Fisher's exact test showed no significant difference between the 2 groups (Table II).

To determine and compare the mean score of sexual satisfaction in women suffering from infertility before the intervention (baseline), after intervention (8 ${}^{\text{th}}$ wk), and follow-up (12 ${}^{\text{th}}$ wk), repeated measure ANOVA and Bonferroni post hoc test were used. The mean score of sexual satisfaction of women suffering from infertility in the intervention group was 62.9 ± 7.32, 71.6 ± 5.95, and 70.9 ± 6.26, before the intervention (baseline), after intervention (8 ${}^{\text{th}}$ wk), and follow-up (12 ${}^{\text{th}}$ wk) respectively. The mean score of sexual satisfaction of women suffering from infertility in the control group was 63.3 ± 6.82, 64.2 ± 7.93, and 62.25 ± 7.99 at baseline, 8 ${}^{\text{th}}$ wk later, and after follow-up (12 ${}^{\text{th}}$ wk, respectively) (Table III).

The results showed that in the intervention group, the mean score of sexual satisfaction improved after the intervention (8 ${}^{\text{th}}$ wk) (p < 0.001) and in the follow-up period (p < 0.001) (12 ${}^{\text{th}}$ wk). But there was no significant difference between the mean score of sexual satisfaction after intervention (8 ${}^{\text{th}}$ wk) and the follow-up period in the intervention group (p = 0.21) (Table IV).

Also, in the control group, the mean of sexual satisfaction in the pretest was 63.3, in the posttest, it was 64.2, and one month later in follow-up it was 62.25 (p = 1.00) (Table V).

**Figure 2:**
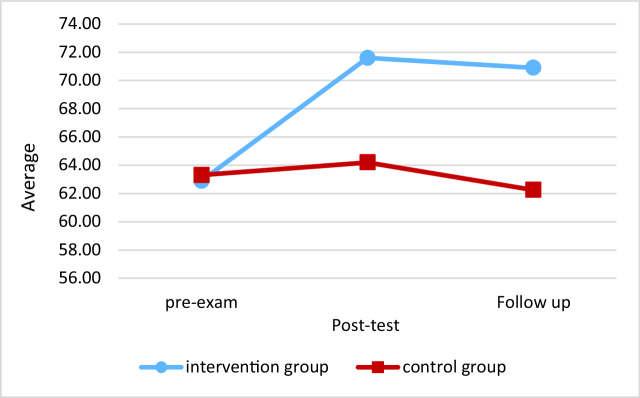
Comparison of mean sexual satisfaction in the intervention and the control group.

No significant difference was observed between the mean of sexual satisfaction in 3 different periods (pretest, post-test, and follow-up) in the control group (p = 0.30). While, a significant difference was observed between the mean of sexual satisfaction before intervention, and (follow-up) in the intervention group (Figure 2).

**Table 2:** Demographic characteristics of women in the intervention and control groups


**Variables**		**Intervention**	**Control**	**P-value**
Woman age*		30.65 ± 4.76	29.45 ± 5.10	0.44 ${}^{\text{a}}$
Number of children*		1.80 ± 0.41	1.85 ± 0.36	0.68 ${}^{\text{a}}$
Duration of marriage*		8.90 ± 4.74	7.70 ± 3.11	0.35 ${}^{\text{a}}$
Duration of infertility (Y)*		7.20 ± 5.26	6.47 ± 3.08	0.59 ${}^{\text{a}}$
Job**				
	**Housewife**	14 (0.70)	12 (0.60)	
	**Employed**	6 (0.30)	8 (0.40)	0.51 ${}^{\text{b}}$
Education**				
	**High school**	6 (0.30)	6 (0.30)	
	**Diploma**	5 (0.25)	6 (0.30)	
	**University**	9 (0.45)	7 (0.40)	0.21 ${}^{\text{b}}$
Fertility type**				
	**Primary**	16 (94.1)	17 (89.5)		
	**Secondary**	4 (5.9)	3 (10.5)	0.60 ${}^{\text{c}}$
	**Female**	7 (0.35)	7 (0.35)		
	**Male**	6 (0.30)	6 (0.3)	
	**Unknown**	7 (0.30)	3 (0.15)	0.37 ${}^{\text{c}}$
History of childbearing**				
	**Yes**	4 (0.20)	3 (0.15)	
	**No**	16 (0.80)	17 (0.85)	0.67 ${}^{\text{c}}$
*Data presented as Mean ± SD. **Data presented as n (%). a: Independent *t* test, b: Chi-square, c: Fisher's exact test				

**Table 3:** Mean score of sexual satisfaction of women suffering from infertility in the intervention and the control group


**Time**	**Intervention group**	**Control group**	*P-value
**Baseline**	62.9 ± 7.32	63.3 ± 6.82	0.300*
**After the intervention (8 ${}^{\text{th}}$ wk)**	71.6 ± 5.95	64.2 ± 7.93	0.001*
**Follow-up (12 ${}^{\text{th}}$ wk)**	70.9 ± 6.26	62.25 ± 7.99	0.001*
**P-value	< 0.001	0.30	
Data presented as Mean ± SD, *Independent *t* test, **Repeated measure ANOVA			

**Table 4:** Mean score of sexual satisfaction of women suffering from infertility in the intervention group in the baseline, 8 ${}^{\text{th}}$ wk, and follow-up period


			<**95% confidence interval mean differences**	
**Test (I)**	**Test (J)**	**Difference**
			**averages (I-J)**	**Criterion error**
**measurement**	*P-value	**Lower limit**	**Upper limit**	
**Baseline **		After the intervention (8 ${}^{\text{th}}$ wk)	-8.7	1.45	0.001	-12.5	-4.89
**Baseline**	follow-up (12 ${}^{\text{th}}$ wk)	-8	1.51	0.001	-11.96	-4.03
**After the intervention** **(8 ${}^{\text{th}}$ wk)**	follow-up (12 ${}^{\text{th}}$ wk)	0.7	0.36	0.21	-0.25	1.65
*Bonferroni posttest						

**Table 5:** Mean score of sexual satisfaction of women suffering from infertility in the control group in baseline, 8 ${}^{\text{th}}$ wk, and follow-up period


			<**95% confidence interval mean differences**	
**Test (I)**	**Test (J)**	**Difference**
			**averages (I-J)**	**Criterion error**
**measurement**	*P-value	**Lower limit**	**Upper limit**	
**Baseline**		After the intervention (8 ${}^{\text{th}}$ wk)	-0.9	1.16	1	-3.96	2.16
**Baseline**	follow-up (12 ${}^{\text{th}}$ wk)	1.05	1.32	1	-2.42	4.52
**After the intervention** ** (8 ${}^{\text{th}}$ wk)**	follow-up (12 ${}^{\text{th}}$ wk)	1.95	1.13	0.3	-1.03	4.93
*Bonferroni posttest results						

## 4. Discussion

This study aimed to determine the effect of sexual health counseling based on mindfulness approaches on sexual satisfaction in women suffering from infertility. The results showed that mindfulness had a significant impact on improving the sexual satisfaction of these women after the intervention and follow-up period.

A qualitative study showed that infertile couples face 4 basic challenges. They include shock, reaction, processing, and reorientation. The subthemes were disbelief and denial, internal processing, avoidance, marriage at risk, external processing, stigma, forgetting, saving marriage, and sexual consent (21).

Many studies recommended sexual health counseling for women suffering from infertility. One study showed that mindfulness-based cognitive therapy reduced anxiety, stress, and depression (9). In addition, a different study recommended health-education program based on the BASNEF model of sexual health satisfaction and satisfaction with quality of sexual relationship among women with infertility (22). Another study suggested sexual counseling based on BETTER model in women with primary infertility and sexual dysfunction (23). In explaining these studies, it can be said that any psychological intervention is associated with improving the sexual health of women who suffer from infertility.

The results of this study were similar to the some studies (24, 25). They showed the effectiveness of mindfulness training on the sexual satisfaction of reproductive-age women (25). The results of another study showed that mindfulness reduced marital stress in women of childbearing age (26). The results of this study are in line with the 2 studies.

Mindfulness improved the quality of life and emotional self-efficacy of women suffering from infertility (27). In addition, mindfulness improved the quality of life of women suffering from infertility in terms of physical, mental, social, and environmental dimensions (28).

The number of mindfulness sessions in the present study was 8 consecutive weekly sessions, but the duration of training in the 2 studies was shorter.

In one study, 2 sessions of sexual counseling based on the BETTER model improved sexual satisfaction of women suffering from infertility (23). This research shows that we can achieve similar results with fewer sex counseling sessions. Especially in our research, the large number of counseling sessions caused the reluctance of some eligible people.

Their research has shown that mindfulness training has an impact on depression and anxiety in women suffering from infertility (29, 30). This method reduces anxiety and meta-worry in women who have recurrent miscarriages (31) and treats depressive symptoms in diabetic patients (32). Infertility causes anxiety, depression, lower self-esteem, lower quality of life, and lower sexual performance of women (33). They showed that mindfulness training reduced the perceived stress of women suffering from infertility and improved irrational cognition in women suffering from infertility who were undergoing IVF treatment (34). The mindfulness training improved/reduced erectile dysfunction in men (35), in men, and improved sexual arousal in women (36). Our study is consistent with the above studies. The results of these studies suggest that the effect of mindfulness counseling can be noted even in the short term. Also, the present study, is similar to that of Farajkhoda study (24). Common feelings of infertility, such as loss, depression, anger, despair, shame, and anxiety, often overshadow the usual feelings of warmth, love, and emotional connection that are the natural conditions of enjoyable sexual relationship (33). Another form of mind training is based on cognitive therapy. At the deepest level, they can dominate their reactions on their own and change their response when dealing with stressful situations, where they can provide positive responses rather than negative ones (11). It leads to success in couple relationships, increased emotional functioning, and stress management (37). Thus, the mindfulness technique, which is a methodological therapeutic approach, can improve sexual satisfaction (38) and arousal disorder (35), and improve sexual behavior by creating positive psychological and psychological effects, including reducing stress and anxiety (25).

Despite the differences between the target group, data collection tools, and counseling approach, the results of studies showed that counseling improved sexual satisfaction, sexual function, stress, quality of life, and depressive symptoms in infertile individuals. In explaining these findings, it can be said that mindfulness consists of a receptive, judgmental consciousness of what is up to the minute. Mindful individuals observe inner and outer realities easily, and can deal with a wide range of opinions, both pleasant and unpleasant emotions. The explanation of this hypothesis implies that women suffering from infertility in the training group are educated by mindfulness-based cognitive therapy by emphasizing the factor of presence in the present and by emphasizing the non-judgmental and purposeful factor. Sharing opinions and correcting misconceptions was one of the most important achievements of women in this study.

## 5. Conclusion 

According to the results of this study, it seems that mindfulness group training in the form of counseling affects the cognitive systems and information processing by increasing people's awareness of how they deal with the psychological effects of infertility. Therefore, considering the effectiveness of this type of counseling and its benefits in improving sexual satisfaction, its widespread use as a method of prevention and non-pharmacological treatment of individuals is recommended. The results of this study suggest that the sexual, physical, and mental health of women suffering from infertility should be given greater importance, as well as health personnel. Midwives, especially, have an important role to play in educating them about the psychological and physical effects associated with infertility and reducing their complications. It is time for sexual satisfaction and marital intimacy not to be compromised and improved.

One of the strengths of the study is the participants' willingness to continue sexual counseling sessions. One of the weak points of this study was not possible to hold online sexual counseling sessions.

### Research limitations

One of the major limitations of this study was the lack of switch over all issues affecting sexual satisfaction, which was partially controlled by random allocation.

Impossibility of long follow-up period.

The unwillingness of some attendees to attend the meetings was due to the lengthy time of classes and the inclusion of those who initially agreed to hold 8 sessions weekly.

Confidence in reporting self-reported mental illness was a limitation of our study.

##  Conflict of Interest 

The authors declare that there is no conflict of interest. 

## References

[B1] Yao H, Chan CHY, Chan CLW (2018). Childbearing importance: A qualitative study of women with infertility in China. Res Nurs Health.

[B2] Feng J, Wang J, Zhang Y, Zhang Y, Jia L, Zhang D, et al (2021). The efficacy of complementary and alternative medicine in the treatment of female infertility. Evid Based Complement Altern Med.

[B3] Rangel EL, Castillo-Angeles M, Easter SR, Atkinson RB, Gosain A, Hu Y-Y, et al (2021). Incidence of infertility and pregnancy complications in US female surgeons. JAMA.

[B4] Petraglia F, Serour GI, Chapron Ch

[B5] Liang Sh, Chen Y, Wang Q, Chen H, Cui Ch, Xu X, et al (2021). Prevalence and associated factors of infertility among 20-49 year old women in Henan province, China. Reprod Health.

[B6] Amiri M, Sadeqi Z, Hoseinpoor MH, Khosravi A (2016). Marital satisfaction and its influencing factors in fertile and infertile women. J Family Reprod Health.

[B7] Abedi Shargh N, Bakhshani NM, Mohebbi MD, Mahmudian Kh, Ahovan M, Mokhtari M, et al (2016). The effectiveness of mindfulness-based cognitive group therapy on marital satisfaction and general health in woman with infertility. Glob J Health Sci.

[B8] Luk BHK, Loke AY (2019). Sexual satisfaction, intimacy and relationship of couples undergoing infertility treatment. J reprod Infant Psychol.

[B9] Marvi N, Golmakani N, Heidarian Miri H, Esmaily H (2019). The effect of sexual education based on sexual health model on the sexual function of women with infertility. Iran J Nurs Midwifery Res.

[B10] Bokaie M, Simbar M, Yassini Ardekani SM (2015). Sexual behavior of infertile women: A qualitative study. Iran J Reprod Med.

[B11] Ashrafian F, Sadeghi M, Rezaei F, Kazemi rezaei SV (2020). [The effect of integrative positive cognitive behavioral therapy on infertility stress and hope in infertile women]. Nurs Midwifery J.

[B12] Zayed AA, El-Hadidy MA (2020). Sexual satisfaction and self-esteem in women with primary infertility. Middle East Fertil Soc J.

[B13] Sahraeian M, Lotfi R, Qorbani M, Faramarzi M, Dinpajooh F, Ramezani Tehrani F (2019). The effect of cognitive behavioral therapy on sexual function in infertile women: A randomized controlled clinical trial. J Sex Marital Ther.

[B14] Bokaie M, Simbar M, Yassini-Ardekani SM (2018). [Social factors affecting the sexual experiences of women faced with infertility: A qualitative study]. Koomesh.

[B15] Kabat-Zinn J (2003). Mindfulness-based stress reduction (MBSR). Constructivism in the Human Sciences.

[B16] Ozturk S, Sut HK, Kucuk L (2019). Examination of sexual functions and depressive symptoms among infertile and fertile women. Pak J Med Sci.

[B17] Nakić Radoš S, Soljačić Vraneš H, Tomić J, Kuna KJ (2022). Infertility-related stress and sexual satisfaction: A dyadic approach. J Psychosom Obstet Gynecol.

[B18] Sadeghi M, Farajkhoda T, Khanabadi M, Eftekhar M (2022). PERMA model vs. integrative-behavioral couple therapy for fertility problems: A randomized clinical trial protocol Int J Reprod BioMed.

[B19] Larson JH, Anderson SM, Holman TB, Niemann BK (1998). A longitudinal study of the effects of premarital communication, relationship stability, and self-esteem on sexual satisfaction in the first year of marriage. J Sex Marital Ther.

[B20] Bahrami N, Yaghoobzadeh A, Sharif Nia H, Soliemani MA, Haghdoost AA (2016). [Psychometric properties of the Persian version of Larsons sexual satisfaction questionnaire in a sample of Iranian infertile couples]. Iran J Epidemiol.

[B21] Aghakhani N, Ewalds-Kvist BM, Sheikhan F, Merghati Khoei E (2020). Iranian women’s experiences of infertility: A qualitative study. Int J Reprod BioMed.

[B22] Shahbazi A, Behboodi Moghadam Z, Maasoumi R, Saffari M, Mohammadi S, Montazeri A (2020). Effect of a health-education program based on the BASNEF model of overall sexual health satisfaction and satisfaction with quality of sexual relationship among women with infertility. Int J Womens Health.

[B23] Karakas S, Aslan E (2019). Sexual counseling in women with primary infertility and sexual dysfunction: Use of the BETTER model. J Sex Marital Ther.

[B24] Farajkhoda T, Ashrafi F, Bokaie M, Zareei Mahmoodabadi H (2021). Online compared to face-to-face sexual intimacy enhancement training program counseling with cognitive-behavioral approach on sexual Intimacy in pregnant women. J Sex Marital Ther.

[B25] Farajkhoda T, Sohran F, Molaeinezhad M, Fallahzadeh H (2019). The effectiveness of mindfulness-based cognitive therapy consultation on improving sexual satisfaction of women in reproductive age: A clinical trial study in Iran. J Adv Pharm Edu Res.

[B26] Bokaie M, Alian FM, Farzinrad B, Dehghani ALI (2018). The effectiveness of group counseling based-mindfulness on marital stress in women of reproductive age: A clinical trial. Int J Phrm Res.

[B27] Mirzaei Moein RM, Saedi S, Razani M (2018). Evaluating the effect of mindfulness-based cognitive therapy on quality of life and emotional self-efficacy in infertile women. Spec J Psychol Manag.

[B28] Rahmani Fard T, Kalantarkousheh SM, Faramarzi M (2017). [The effect of mindfulness-based cognitive psychotherapy on quality of life in infertile women]. Hayat.

[B29] Rooney KL, Domar AD (2018). The relationship between stress and infertility. Dialogues Clin Neurosci.

[B30] Ordoni Avval Z, Rabiepoor S, Behroozilak T, Arefi M, Yas A (2019). The effectiveness of counseling with a cognitive-behavioral approach on infertile women’s stress. Maedica.

[B31] Sharifi-Shaki S, Aakhte M, Alipor A, Fahimi-Far A, Taghadosi M, Karimi R, et al (2015). [The effectiveness of mindfulness-based cognitive therapy in reducing anxiety and meta-worry in women with recurrent miscarriages]. Feyz.

[B32] Tovote KA, Fleer J, Snippe E, Peeters AC, Emmelkamp PM, Sanderman R, et al (2014). Individual mindfulness-based cognitive therapy and cognitive behavior therapy for treating depressive symptoms in patients with diabetes: Results of a randomized controlled trial. Diabetes Care.

[B33] Kulaksiz D, Toprak T, Ayribas B, Ozcan E, Arslan U, Dokuzeylul Gungor N (2022). The effect of male and female factor infertility on women’s anxiety, depression, self-esteem, quality of life and sexual function parameters: A prospective, cross-sectional study from Turkey. Arch Gynecol Obstet.

[B34] Wang X, Wang Y

[B35] Bossio JA, Basson R, Driscoll M, Correia S, Brotto LA (2018). Mindfulness-based group therapy for men with situational erectile dysfunction: A mixed-methods feasibility analysis and pilot study. J Sex Med.

[B36] Brotto LA, Zdaniuk B, Chivers ML, Jabs F, Grabovac AD, Lalumière ML (2022). Mindfulness and sex education for sexual interest/arousal disorder: Mediators and moderators of treatment outcome. J Sex Res.

[B37] O’Kelly M, Collard J, Vernon A Cognitive and rational-emotive behavior therapy with couples.

[B38] Brotto LA, Seal BN, Rellini A (2012). Pilot study of a brief cognitive behavioral versus mindfulness-based intervention for women with sexual distress and a history of childhood sexual abuse. J Sex Marital Ther.

